# MicroRNA-144-3p inhibits bone formation in distraction osteogenesis through targeting Connexin 43

**DOI:** 10.18632/oncotarget.20984

**Published:** 2017-09-18

**Authors:** Yu-Xin Sun, Jin-Fang Zhang, Jia Xu, Liang-Liang Xu, Tian-Yi Wu, Bin Wang, Xiao-Hua Pan, Gang Li

**Affiliations:** ^1^ Department of Orthopaedics and Traumatology, Bao-An District People's Hospital, Shenzhen, PR China; ^2^ Department of Orthopaedics & Traumatology, Li Ka Shing Institute of Health Sciences and Lui Che Woo Institute of Innovative Medicine, Faculty of Medicine, The Chinese University of Hong Kong, Prince of Wales Hospital, Shatin, Hong Kong SAR, PR China; ^3^ The CUHK-ACC Space Medicine Centre on Health Maintenance of Musculoskeletal System, The Chinese University of Hong Kong Shenzhen Research Institute, Shenzhen, PR China; ^4^ Department of Orthopaedics Surgery, Shanghai Jiao Tong University Affiliated Sixth People's Hospital, Shanghai, PR China; ^5^ Key Laboratory for Regenerative Medicine, Ministry of Education, School of Biomedical Sciences, Faculty of Medicine, The Chinese University of Hong Kong, Hong Kong SAR, PR China

**Keywords:** distraction osteogenesis, mesenchymal stem cells, microRNAs, miR-144

## Abstract

Distraction osteogenesis (DO), one of effective therapies for bone regeneration, has been received more attention in recent years. However, the underlying mechanism remains elusive. Recently, microRNAs (miRNAs) have been reported to play important roles in regulating osteogenesis and bone formation. We therefore provided the hypothesis that miRNAs could involve in the DO-mediated bone regeneration. After successfully established the DO model of rats, a miRNA microarray was performed to find the differently expressed miRNAs in DO and control groups in this study. As one of the most downregulated miRNAs, miR-144-3p was found to be decreased during osteogenic differentiation in mesenchymal stem cells of rats (rBMSCs) and DO model. And miR-144-3p overexpression suppressed the osteogenesis while its inhibitor promoted osteogenesis. Furthermore, Connexin-43, an essential regulator for osteogenesis, was validated to be a novel target for miR-144-3p. Finally, miR-144-3p inhibitor modified MSCs promoted mineralization of distracted bone in rat DO model. In conclusion, miR-144-3p was found to regulate osteogenesis and inhibition of miR-144-3p resulted in acceleration of mineralization of DO, which not only give clues to understanding the mechanism of DO but also provide a potential therapeutic target in clinical practice.

## INTRODUCTION

Distraction osteogenesis (DO) is an efficiency approach to promote bone formation in clinical practice. Newly formed bone could be generated gradually in the distraction gap, along with the application of regular tensile forces [1]. However, the underlying mechanism of DO to promote bone formation remains elusive [2]. Additionally, the delayed consolidation of distracted bone is one of the major disadvantage of DO which would limit its application in clinics [3]. Therefore, there is a burning need to develop a new strategy to accelerate bone regeneration during the DO process.

As novel regulators in bone regeneration, miRNAs have been reported to play important roles in skeletal development and disorders. For example, evidence indicate that let-7, miR-140 and miR-92a are crucial for skeletal development [4–5]; and deficiency of these miRNAs suppress the proliferation as well as the differentiation of growth plate chondrocytes, leading to a dramatic growth defect [4–5]. Besides, the expression of some critical miRNAs also reflects the progress of bone related diseases. For instance, miR-9, -27, -34a, and -140 were found aberrantly expressed in osteoarthritis patients [6], while miR-21, -23a, -24, -100, and -125b were significantly increased both in serum and bone tissues of osteoporosis patients [7]. These findings indicated miRNAs not only involved in the pathological process of skeletal disease but also could be served as potential markers for osteoarthritis and osteoporosis. Moreover, our previous study found that miR-21 modified mesenchyme stem cells (MSCs) could promote fracture healing of rats [7]. Li *et al* also reported local delivery of miR-26a into the defected bone of nude mice could promote bone regeneration [8]. Chen *et al* showed that overexpression of miR-503 could inhibit bone resorption and prevent bone loss in osteoporosis mice [9]. These studies demonstrated that miRNAs not only contribute to bone formation but also could be acted as a novel therapy for severe bone disorders.

Till now, few miRNAs have been reported to be involved in DO process except miR-503 which promoted bone formation in distraction osteogenesis [10]. In this study, another miRNA, miR-144-3p was identified as a crucial miRNA during DO process. Our results demonstrated that miR-144-3p suppressed osteoblast and bone formation in distraction osteogenesis, therefore miR-144-3p intervention therapy would be a promising strategy for bone regeneration of DO.

## RESULTS

### miR-144-3p was downregulated in bone tissues with DO

**Figure 1:**
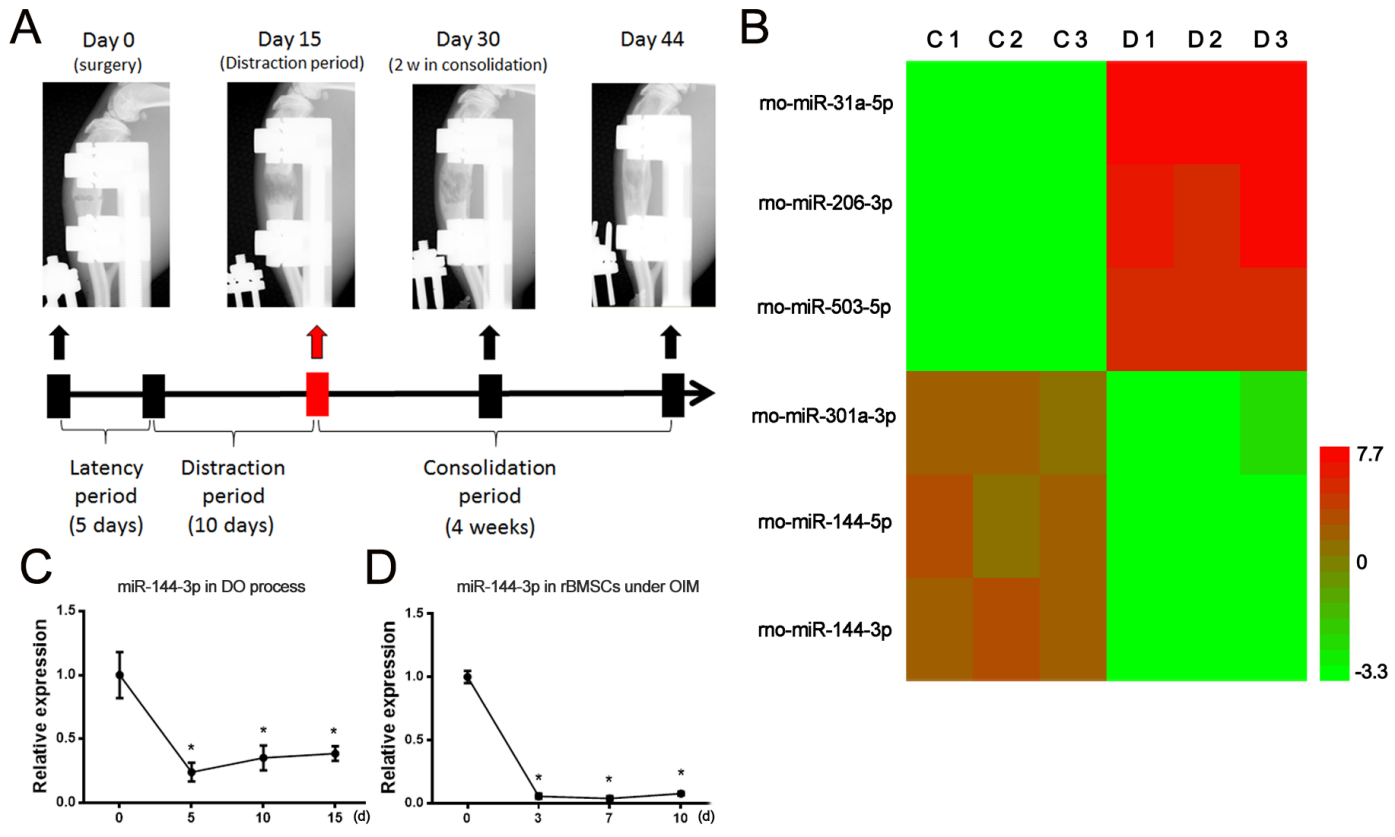
MiR-144-3p was downregulated during DO process and osteogenic differentiation **(A)** DO model in rats. Bone formation in the distraction gap was detected by X-ray. **(B)** At the end of distraction phase, bone tissue from distraction gap were harvested for a microRNA microarray test. The contralateral tibiae were also harvested for normal control (n=3 per group). Heatmap of microarray results (Simplified version) was shown, and the top three up/down regulated microRNAs were listed. Red color represents up regulation while green color represents down regulation. C: control group; D: distraction group (n=3 each group). **(C)** Expression of miR-144-3p during DO process. Fold change of miR-144-3p expression level at different time points were calculated through compared to the expression level at day 0. (n=3 each time points, ^*^ *P*<0.05 compared to Day 0, U6 was used as an internal reference). **(D)** Expression of miR-144-3p during osteogenic differentiation of rBMSCs. Osteogenic induction was performed and cells were harvested at day 0, 3, 7 and 10. Fold change of miR-144-3p expression at different time points were calculated through compared to the expression level at day 0. (n=3 each time points, ^*^ *P*<0.05 compared to Day 0).

After successfully established the DO model of rats, the bone tissues of distraction gap were harvested at the end of distraction period for a miRNA microarray assay (Figure 1A). According to the heat map results, miR-144-3p was found to be the most downregulated miRNAs (Figure 1B). To verify the results of microarray, Quantitative polymerase chain reaction (qPCR) was performed to detect the expression level of miR-144-3p during DO process and the results showed that it was significantly downregulated during the distraction period of DO (Figure 1C). We further examined the expression of miR-144-3p during the osteogenic differentiation of rat MSCs derived from bone marrow (rBMSCs), and the results showed that it also was decreased during the osteogenic differentiation of rBMSCs (Figure 1D). Taken together, the downregulation of miR-144-3p would play important roles in regulating the calcification in DO and osteogenic differentiation of rBMSCs.

### MiR-144-3p suppressed while its inhibitor promoted osteogenic differentiation of rBMSCs

**Figure 2:**
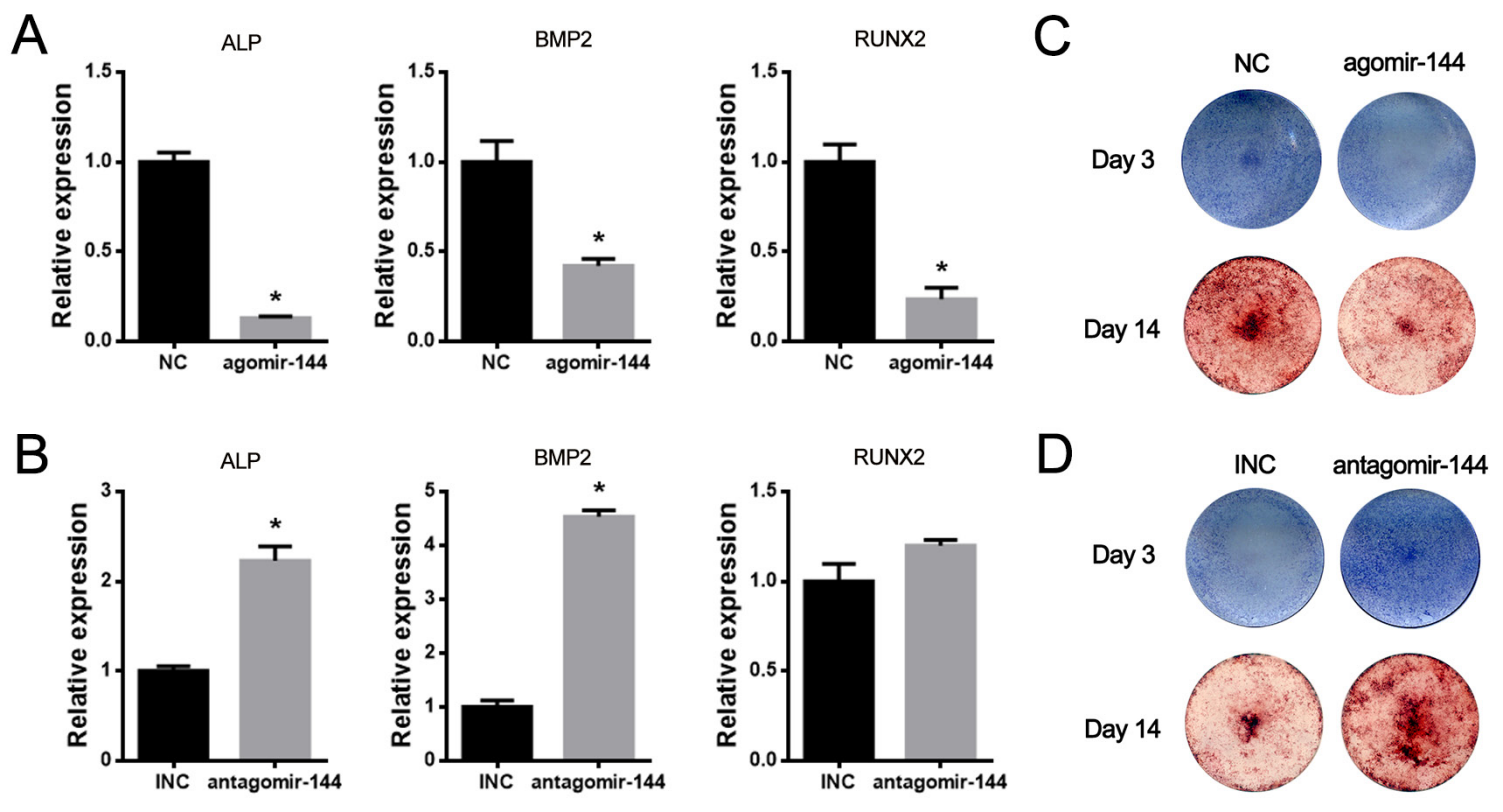
MiR-144-3p suppressed while its inhibitor promoted osteogenic differentiation of rBMSCs **(A&B)** Expression of osteogenic markers after agomir & antagomir-144 transfection in rBMSCs. Expressions of ALP and BMP2 were increased after antagomir-144 transfection in rBMSCs under osteogenic differentiation, while expressions of ALP, RUNX2 and BMP2 were decreased after agomir-144 transfection. (n=3, ^*^ *P*<0.05, GAPDH was used as an internal reference) **(C-D)** ALP and Alizarin red staining. Results of ALP & Alizarin red staining were enhanced by antagomir-144 and reduced by agomir-144 transfection in rBMSCs under osteogenic differentiation.

To elucidate the functional characterization of miR-144-3p on osteogenesis, agomir-144 (miR-144-3p mimics) and antagomir-144 (miR-144-3p inhibitor) were transiently transfected into rBMSCs and the osteogenic effects were examined. As shown in Figure 2A&2B, the osteogenic marker genes such as ALP, BMP2 and Runx2 were downregulated by agomir-144, while they were upregulated by antagomir-144. The further staining results of ALP and Alizarin red also demonstrated that miR-144-3p suppressed while its inhibitor could promote osteogenic differentiation (Figure 2C–2D).

### Connexin 43 was a real target for miR-144-3p

**Figure 3:**
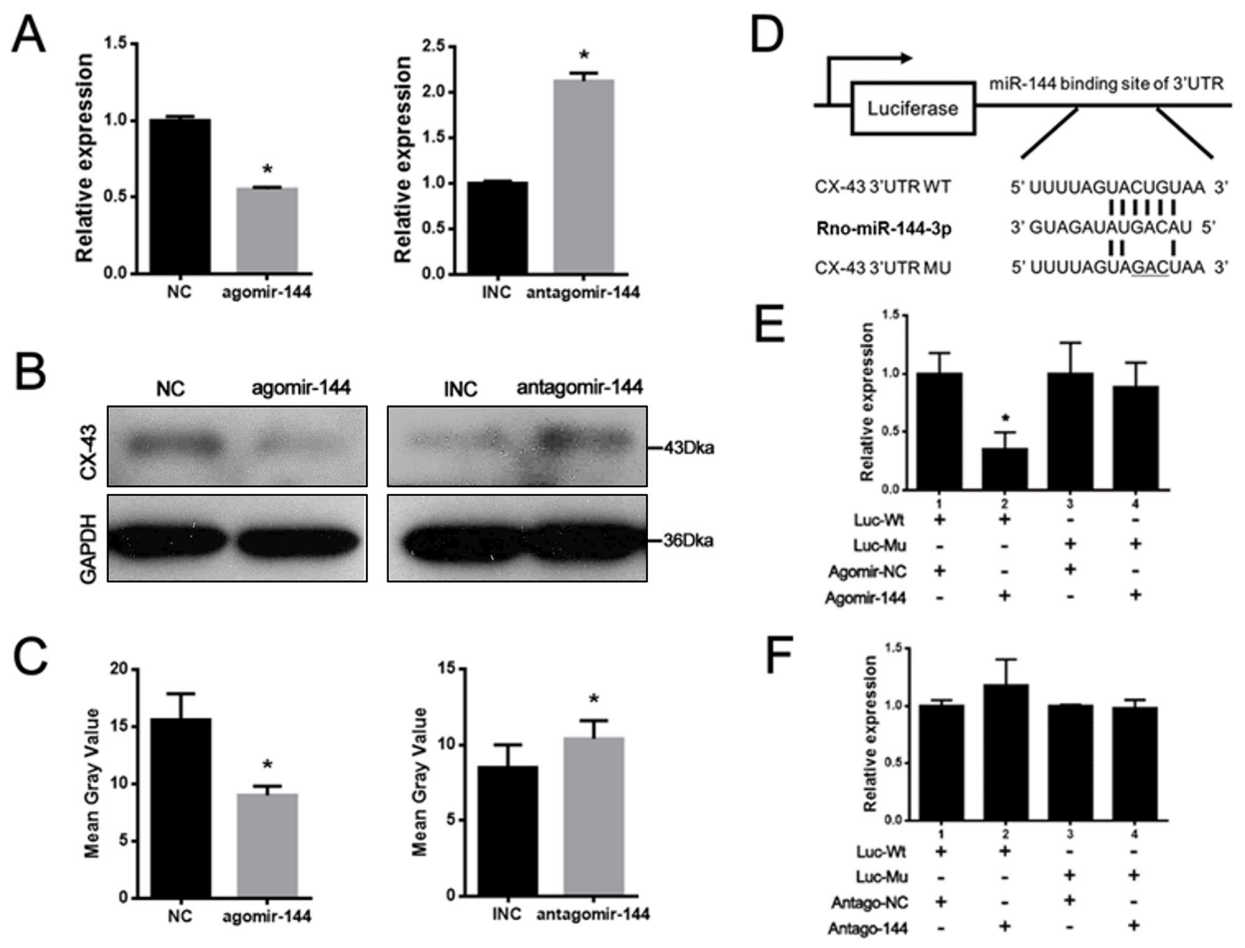
CX-43 was a real target gene of miR-144-3p **(A)** Expression of CX-43 mRNA after agomir & antagomir-144 transfection in rBMSCs. CX-43 mRNA expression was decreased after agomir-144 transfection and increased after antagomir-144 transfection. (n=3, ^*^ *P*<0.05, GAPDH was used as an internal reference) **(B)** Western blotting assays. CX-43 protein expression was decreased after agomir-144 transfection and increased after antagomir-144 transfection by western blotting. **(C)** The bands were quantified by the mean gray value with Image J software. Significant difference in gray value could be found after agomir-144/antagomir-144 transfected, compared to the NC/INC group. (n=3, ^*^ P<0.05, GAPDH was used as an internal reference). **(D)** Illustration of luciferase reporters. Wild type (Wt) and mutant (Mu) binding sites in 3′ UTR of CX-43 were designed and were inserted into the luciferase reporter. (E&F) Luciferase assays. The luciferase reporters and agomir-144 and antagomir-144 were co-transfected into 293T cells to perform luciferase assay. Result showed that firefly/Rennila ratio was decreased when agomir-144/ antagomir-144 and luciferase reporter containing Wt 3′ UTR of CX-43 were co-transfected into 293T cells, while no difference was found in agomir-144/ antagomir-144 co-transfected with Mu 3′ UTR of CX-43. (n=3, ^*^ P<0.05).

Based on the prediction by bioinformatics analyses, Connexin 43 (CX-43) is a promising candidate with potential binding sites in its 3′-UTR for miR-144-3p. With agomir-144 transfection, the mRNA and protein levels of CX-43 were both significantly reduced in rBMSCs (Figure 3A–3C). To further validate whether CX-43 was a bona fide target for miR-144-3p, the binding and the mutated sites into the 3′ UTR of CX-43 were inserted into the pmiR-Glo vector (Figure 3D). It was shown that miR-144-3p dramatically suppressed the luciferase activity when compared with control groups and mutations on these binding sites successfully abolished the suppressive effects (Figure 3E–3F). These results suggest that CX-43 is a real target for miR-144-3p in rBMSCs.

### MiR-144-3p inhibitor attenuated the suppressive effect of siCX-43 on osteogenesis

**Figure 4:**
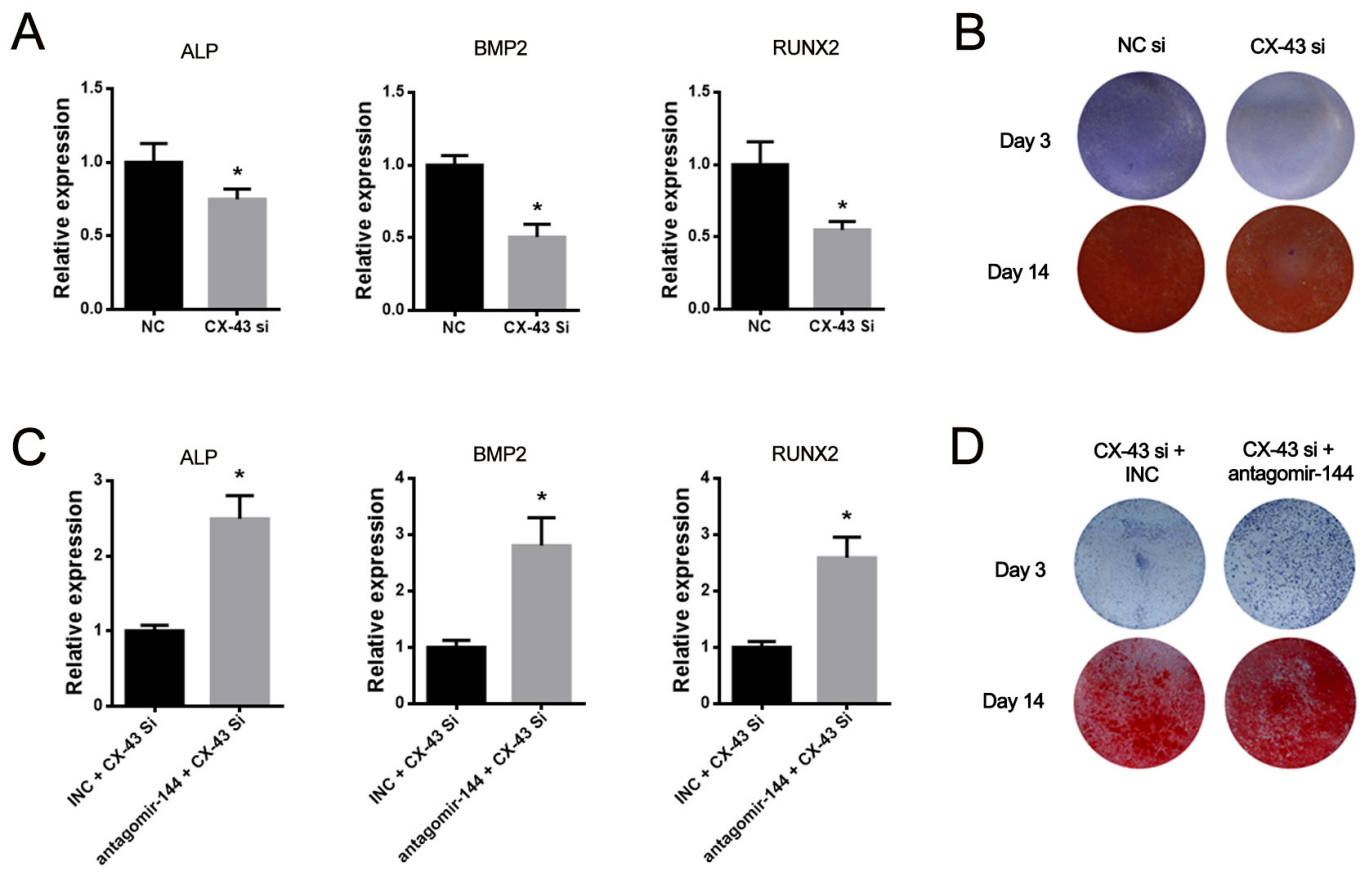
MiR-144-3p inhibitor attenuated the suppression of osteogenesis induced by CX-43 knockdown **(A)** Expression level of osteogenic markers after CX-43 silenced by siRNA. Significant downregulation of ALP, RUNX2 and BMP2 expression was observed by siCX-43. **(B)** ALP and alizarin red staining after siCX-43 treatment. Results of staining showed that osteogenic effect was suppressed by siCX-43. **(C&D)** Rescue experiments. Co-transfection of CX-43 siRNA and antagomir-144 increased the expression levels of ALP, RUNX2 and BMP2. The co-transfection also increased the staining outcomes of ALP & Alizarin Red staining. (n=3, ^*^ *P*<0.05, GAPDH was used as the internal reference).

To elucidate whether miR-144-3p mediates osteogenesis *via* suppressing CX-43 expression, a small interfering RNA (siRNA) specifically targeting CX-43 (siCX-43) was designed and its osteogenic effect was investigated. The expression of osteogenic marker genes such as ALP, BMP2 and RUNX2 were suppressed by siCX-43 (Figure 4A). The further ALP and Alizarin red staining also confirmed that CX-43 knockdown suppressed the osteogenesis of rBMSCs (Figure 4B). Furthermore, we also found that the antagomir-144 partially reversed the downregulation of marker genes (Figure 4C) and rescued the suppressive effect on osteogenesis induced by siCX-43 (Figure 4D). Collectively, these data indicated that miR-144-3p could regulate osteogenesis through suppressing CX-43 expression.

### Local injection of miR-144-3p inhibitor modified rBMSCs accelerated bone formation in DO animal model

**Figure 5:**
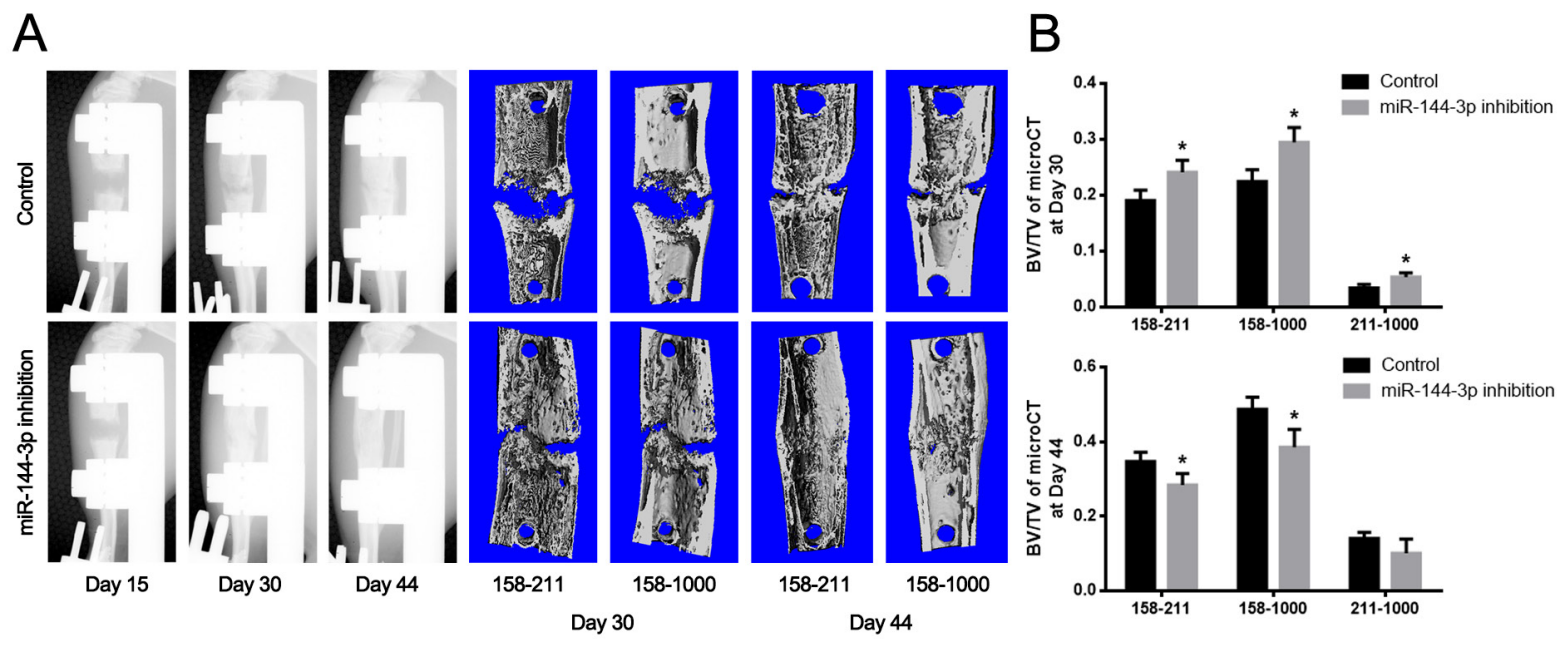
Detection of the newly formed bone by X-ray and microCT examination **(A)** Imageological examination. X-ray examination was performed at day 15, 30 and 44. Series of representative radiography were shown. The structure of the distracted bone had already repaired as normal at day 44 postoperation in miR-144-3p inhibition group. An obvious crack in the middle of distraction gap still could be found in control group. At 44 days post-operation, the central 150 layers bone tissues within the distraction gap were analyzed by microCT examination. (Vertical interval between red dotted lines represented the distraction gap). **(B)** Quantitative analysis by microCT. Result showed BV/TV within 158-211 as well as 158-1000 were much higher at day 30 but much lower at day 44 post-operation in miR-144-3p inhibition group. (n=10 each group, ^*^ *P*<0.05).

**Figure 6:**
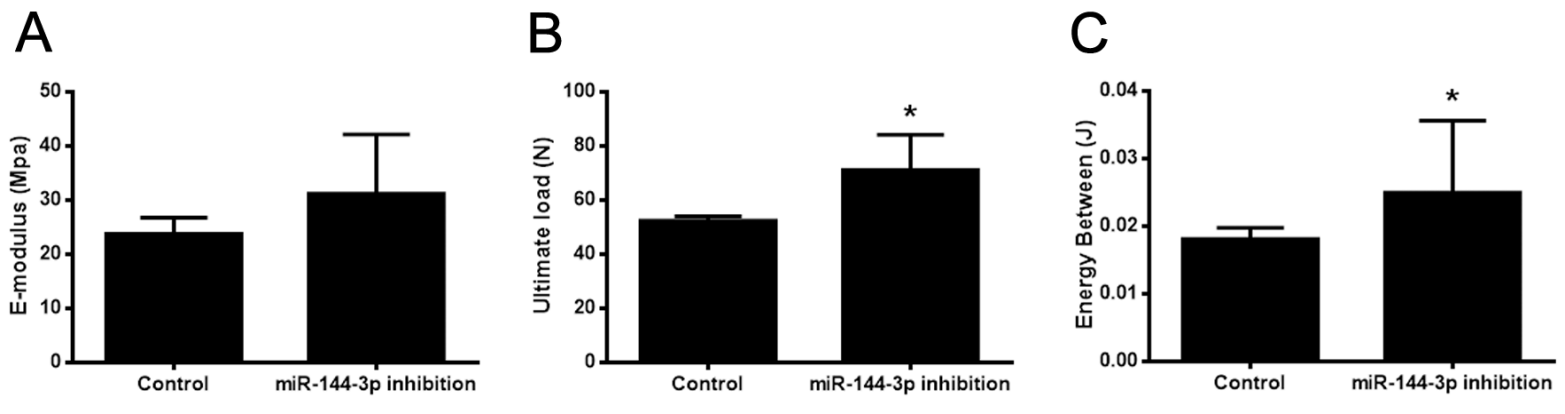
Bone healing property was enhanced by miR-144-3p inhibitor therapy Mechanical test was performed on the lengthened tibia after all the animals sacrificed at day 44 post-operation. Form the results, no significant differences were found in E-modulus **(A)** between these two groups. However, ultimate load **(B)** and energy between **(C)** were much higher in miR-144-3p inhibition group than that in control group (n=10 each group, ^*^ *P*<0.05).

**Figure 7:**
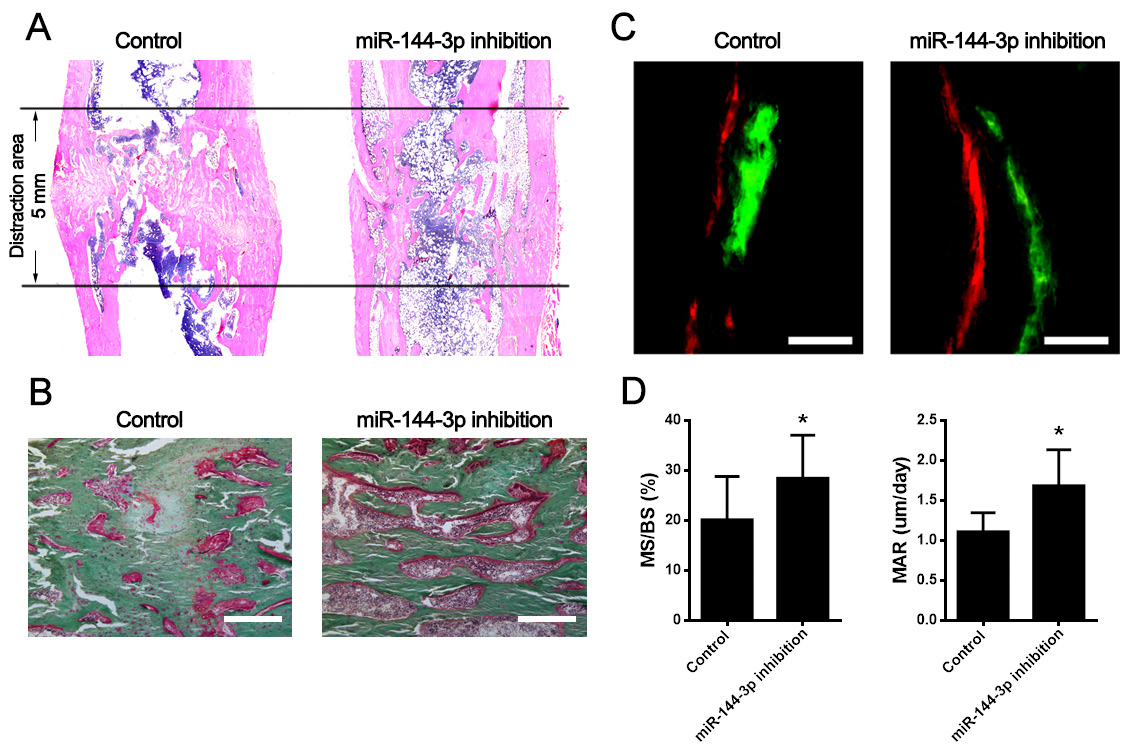
Bone tissue regeneration was accelerated by miR-144-3p inhibitor modified MSCs in DO animal model **(A)** HE analyses of lengthened tibia in sagittal section at day 44 post-operation. In miR-144-3p inhibitor group, less bone tissues were detected in the distraction gap, but the medullary cavity was almost recanalized. In the control group, lots of immature bone tissues and cartilaginous callused still could be found in the distraction gap. **(B)** Goldner's trichrome staining. Result showed that bone remodeling was robust in miR-144-3p inhibition group while lots of regenerated cartilage cells still could be found in the distraction area. (Scale Bar=150 μm) **(C)** Double label staining. Calcein (green) and Xylenol (orange) were subcutaneously injected into the rats at day 15 and day 43 separately. Vertical interval of the lines represented the amount of newly formed bone between the injection interphase. Result indicated miR-144-3p inhibition could promote mineralization of newly formed bone. (Scale Bar=100 μm) **(D)** Quantitative analysis of MAR and MS/BS. The dynamic histomorphometric parameters including MAR and MS/BS were found higher in miR-144-3p inhibition group than that in control group. (n=5 each group, ^*^ *P*<0.05).

To test the therapeutic effect of antagomir-144 on bone regeneration during DO process, stable anti-miR-144-3p modified rBMSCs were established and locally injected into the distraction gap at the end of distraction period. 2 and 4 weeks after injection, distracted bone were harvested for microCT assays and the results showed that miR-144-3p inhibitor stimulated bone formation at week 2 and week 4 (Figure 5A). Furthermore, the rats with anti-miR-144-3p modified MSCs injection displayed a significant increase in total bone volume (BV)/total tissue volume (TV) at week 2, whereas a slight decrease was observed at week 4 (Figure 5B). However, tibiae of rats treated with anti-miR-144-3p modified rBMSCs exhibited better mechanical properties than the control group in ultimate load and energy (Figure 6A–6C). Histological analyses indicated that bone structure was almost recovered in anti-miR-144-3p group, while still a lot of immature callus could be found in the control group (Figure 7A–7B). The quantitative histomorphometry results also demonstrated that higher MAR and MS/BS could be observed in anti-miR-144-3p group compared to the control group (Figure 7C–7D).

## DISCUSSION

As a technique in orthopedics surgery, DO provides a way for reconstruction of skeletal deformities in clinics. To better understand the molecular mechanism of DO, a panel of miRNAs was screened by a microarray analysis, and miR-144-3p was identified as one of the most downregulated miRNAs by DO. In the present study, miR-144-3p was demonstrated to suppress osteogenic differentiation *in vitro*, whereas anti-miR-144-3p promoted osteoblast *in vitro* and accelerated bone formation *in vivo*.

MiR-144 is a family of microRNA precursors found in mammals, including humans. Increasing evidence have demonstrated that miR-144 could be identified as regulator of tumorigenesis. For example, miR-144 was significantly downregulated in esophageal carcinoma [11] and osteosarcoma [12] while overexpressed in pulmonary tuberculosis [13], renal cell carcinoma [14], and medulloblastoma [15]. MiR-144 also reported to activate Wnt signaling in bladder cancer *via* targeting EZH2 [16]. However, the studies on the association between miR-144 and osteogenesis remains limited. Kang *et al* showed that overexpression of miR-144 would inhibit the osteogenic process by targeting Cadherin-11 [17]. Our results also indicated that miR-144-3p suppressed while its inhibitor promoted osteogenic differentiation of rBMSCs.

It is well known that miRNAs mediate biological activities through suppressing target gene expression. Bioinformatic prediction and biological confirmation demonstrated that CX-43 was a real target for miR-144-3p. As the most abundant member of the gap junctional intercellular communication family, CX-43 is essential for embryonic bone development [18] and bone regeneration [19]. The deficiency of CX-43 would result in the delayed ossification [20], enhanced osteoclastic resorption [21] and undesirable effect on fracture healing [22]. Additionally, CX-43 was extremely sensitive to mechanical stimulation in osteocytes [23] and MSCs [24]. Li *et al* found that CX-43, together with RUNX2 and Osterix, was significantly upregulated when cyclic mechanical tension was loaded [25], suggesting the increasing CX-43 expression could be benefit to osteogenesis, which was consistent with our data. In this study, we confirmed that miR-144-3p was significantly downregulated in distraction phase of DO with tensile stimulation. And it was also declined during the osteogenic differentiation of rBMSCs. Further biological function studies also showed that miR-144-3p inhibitor could contribute to osteoblast *in vitro* and bone formation *in vivo* via promoting CX-43 expression. These findings may provide a possibility to explain the underlying mechanism of DO from the point of miRNA regulation.

More importantly, the bone regeneration ability of miR-144-3p inhibitor was also examined in DO animal model. According to the results of mechanical testing and bone histomorphometry, bone healing property was enhanced after treated with anti-miR-144-3p modified rBMSCs. However, the results from microCT assays revealed that there was a significant increase at week 2 but an obvious reduction at week 4 in BV/TV with anti-miR-144-3p injection. It is well-known that the regenerated bone needs to be remodeled at the end of consolidation phase [26]. During this period, most of the regenerated woven bone would be replaced by mature lamellar bone and the distracted bone would be reshaped into its original appearance. The hematein & eosin (HE) staining results confirmed that medullary cavity has been recanalized in miR-144-3p inhibitor group, while lots of immature woven bone still could be found within the distraction gap in control group. Therefore, the rapid bone repair would contribute to the reduction of BV/TV at week 4 in miR-144-3p inhibitor group. All these results demonstrated that the bone healing process was accelerated by miR-144-3p inhibitor therapy.

In conclusion, miR-144-3p was significantly downregulated during DO process and the anti-miR-144-3p could enhance osteogenesis *in vitro* and accelerate bone regeneration *in vivo* through targeting CX-43. The knowledge generated from this study improves our understanding of the bone regeneration of DO and explore the potential of miR-144-3p as a promising therapeutic target for bone fracture in clinical practice. Therefore, a combined strategy between distraction surgery and miRNA adminstration is provided to enhance bone formation, which may shed light on the development for a potential therapeutic target for bone repair.

## MATERIALS AND METHODS

### Animal species and ethics approval

Three-month old Sprague-Dawley male rats were obtained from the Laboratory Animal Research Centre of The Chinese University of Hong Kong (n=1 for cell harvest; n=3 for microarray; n=10 specimens per group). This study was specifically approved by the Animal Experimentation Ethics Committee of the Chinese University of Hong Kong (AEEC No. 14-052-MIS), and carried out under the animal license issued by the Hong Kong SAR Government. All methods were performed in accordance with the relevant guidelines and regulations. All efforts were made to minimize the number of animals used and their suffering.

### Surgical procedure of DO

All the animals were operated under general anesthesia by ketamine (40 mg/kg) and xylazine (4 mg/kg) given intraperitoneally. Mid-diaphyseal corticotomy was created and a custom made external distraction device was fixed to the tibia by four stainless steel pins. Wounds then were closed in layers and animals were free to move in the cage after the surgery.

### Distraction protocol

Rats distraction were performed according to previous reports [27]. After a 5-day latency period, lengthening was initiated at a rate of 0.25 mm/12 hours for 10 days. Then bone segments were maintained the position with external device for another 4 weeks before the animals were sacrificed.

### Sample collection and total RNA extraction

Bone tissues were harvested from distraction gap at the end day of distraction period and immediately thrown into the liquid nitrogen in case of RNA degradation. Then the samples were ground up in RNase-free mortars with liquid nitrogen and TRIzol reagent (Invitrogen, USA) was used to extract the total RNA from the precipitate.

### MiRNA microarray

An Agilent rat miRNA microarray (8^*^60K) was used for global scanning of miRNA expression in total RNA samples. Sample labeling, microarray hybridization, and washing were performed based on the manufacturer's standard protocols (Agilent Technologies Inc., Santa Clara, California, USA). Briefly, total RNA was dephosphorylated, denatured, and then labeled with Cyanine-3-CTP. After purification, labeled RNAs were hybridized onto the microarray. After washing, the arrays were scanned with an Agilent Scanner G2505C (Agilent Technologies Inc., Santa Clara, California, USA). Feature Extraction software (version 10.7.1.1; Agilent Technologies Inc., Santa Clara, California, USA) was used to analyze microarray images and obtain raw data. Next, GeneSpring software (version 12.5; Agilent Technologies Inc., Santa Clara, California, USA) was used to complete the basic analysis using raw data. The raw data was normalized with the quantile algorithm. If the probes with a positive normalized expression value were flagged as “Detected” in at least 100% of samples, they were chosen for further analysis. Differentially expressed miRNAs were then identified through fold change as well as the p value calculated using a Student's t-test. The threshold set for significantly up- and down-regulated genes was a fold change >2.0 and a P value <0.05. The miRNA microarray assay was performed by Shanghai OE Biotech Technology Co, Ltd. (Shanghai, China).

### Cell culture

The rBMSCs were isolated as previously described [28]. Briefly, the rBMSCs were obtained from the bone marrow of 4-week old SD rat and cultured in a 100 mm cell culture dish in the alpha complete culture medium at 37°C with 5% CO_2_ and 95% humidity. The rBMSCs from passages 3-5 were used in the experiments. The surface antigens of rBMSCs were detected by flow cytometry using CD90, CD44, CD34 and CD45.

### Transfection of mimic/inhibitor

The agomir-144 and antagomir-144 oligoes were synthesized from Genepharma Company (Genepharma, China). The transfection of agomir-144 and antagomir-144 were performed with Lipofectamine 2000 (Invitrogen, USA) according to our previous study [29]. The MSCs were seeded into 12 well plates until the cells were transfected with agomir-144 or antagomir-144 oligoes at a concentration of 50 pmol/well (for agomir) or 100 pmol/well (for antagomir).

### Osteogenic differentiation and ALP/Alizarin Red S staining

Briefly, the culture medium was removed and replaced by the osteogenic induction medium (1nM dexamethasone, 50mM L-ascorbic acid-2-phosphate and 20mM β-glycerolphosphate with complete medium). The induction medium was changed every three days during the osteogenic differentiation. Three days and fourteen days after the induction, ALP and Alizarin Red S staining were performed separately to evaluate the alkaline phosphatase activity and calcium deposit formation.

### Quantitative Real-Time PCR for osteomarkers and miR-144-3p

Total RNA from cells extracted with TRIzol Reagent. For osteomarkers assays, complementary DNAs (cDNAs) was synthesized with M-MLV Reverse Transcriptase (Invitrogen) according to the manufacturer's instructions. For miRNAs assays, the cDNAs were generated with All-in-One miRNA quantitative reverse transcription- (qRT-) PCR detection kit (GeneCopoeia, Guangzhou, China). Real-time PCR was performed using the Step One Plus Real-Time PCR System (Applied Biosystems, USA). And primer sequences were as follows: BMP2 forward: 5′ aaggcaccctttgtatgtgg 3′, reverse: 5′ catgccttagggattttgga 3′; RUNX2 forward: 5′ ccgatgggaccgtggtt 3′, reverse: 5′cagcagaggcatttcgtagct 3′; ALP forward: 5′ tccgtgggtcggattcct 3′, 5′ gccggcccaagagagaa 3′. Primer sequences were as follows: miR-144-3p 5′ tacagtatagatgatgtact 3′; U6 forward 5′ ctcgcttcggcagcaca 3′, reverse 5′ aacgcttcacgaatttgcgt 3′. The relative quantification of gene expression was analyzed with 2^-ΔΔCT^ method, normalized with GAPDH (for osteomarkers) of U6 (for miRNA) expression as previously reported [30].

### Target gene prediction and luciferase report assays

Target genes of miR-144-3p were predicted using online programs: TargetScan (http://www.targetscan.org/) and Diana lab (http://diana.imis.athena-innovation.gr/). CX-43 was predicted by both programs. A fragment of the CX-43 3′ UTR containing the predicted binding site or their mutated binding sites were then synthesized and cloned into downstream of the Luciferase reporter gene (CX-43 Wt-Luc or CX-43 Mt-Luc). These plasmids, along with Relina vector and agomir-144 or negative control, were transfected into 293T cells using Lipofectamine 2000. Cells were harvested at 28-30h and luciferase activities were detected using the Dual-Luciferase Reporter Assay System (Promega, USA). The relative luciferase activity value was achieved against the Renilla control as our previously reported [31].

### Western blot analysis

Cell lysates were lysed by RIPA buffer (Sigma-Aldrich, USA) and separated by SDS-PAGE (10%) and transferred to PVDF membranes (Bio-rad). After blocked with 5% skimmed milk for 1 hour, the membrane was probed with the primary antibody CX-43 (1:100, Santa Cruz, USA) and the blots were visualized by the chemiluminescence (ECL, USA). GAPDH (Santa Cruz) was used as the internal control.

### Stable anti-miR-144-3p modified rBMSCs

The rno-miR-144-3p inhibitor lentivirus was purchased from Genepharma Company (Genepharma, China). For infection, 1 × 10^5^ cells were seeded into 6-well plate and incubated with lentiviruses and 8 *μ*;g/mL polybrene in the incubator for 24h according to previous protocol [32].

### Local injection therapy

Mesenchymal stem cells modified by miRNAs intervention were locally injected into the distraction gap percutaneously and monitored by X-ray. All the injections were given on the first day of consolidation. At the end of experiment, all animals were sacrificed and the tibial specimens were harvested for further analysis.

### Micro-CT examination

MicroCT analysis was performed for each animal after sacrificed. Briefly, all the specimens were imaged using a vivaCT 40 (Scanco Medical) with a voltage of 70 keV, a current of 114 *μ*A, and 10.5 *μ*m isotropic resolution. To eliminate the interference by the native bone, the central 150 layers in horizontal plane of the distraction bone were selected as the region of interest. Low- and high-density mineralized tissues were reconstructed using different thresholds (low attenuation = 158, high attenuation = 211) using our established evaluation protocol with small modification. The high-density tissues represented the newly formed highly mineralized calluses and the old cortices, while the low-density tissues represented the newly formed calluses. Bone volume (BV), tissue volume (TV), and BV/TV of each sample were recorded for analysis.

### Fourt-point bending mechanical testing

Mechanical test was performed within 24 hours after sacrificed under room temperature. A four-point bending device (H25KS; Hounsfield Test Equipment Ltd. UK) with a 200N load cell was used to test the distracted tibiae to failure. The tibiae were loaded in the anterior-posterior direction with the inner and outer span of the blades set as 8 and 20 mm, respectively. The long axis of the tibia was oriented perpendicular to the blades during the test. The ultimate load, the energy to failure, and *E*-modulus were recorded and analyzed using built-in software (QMAT Professional; Tinius Olsen, Inc., Horsham, PA, USA).

### Histological analysis

The femora were fixed in 10% buffered formalin, decalcified with 9% formic acid, and embedded in paraffin. Attempts were made to standardize the sectioning at a midsagittal plane of each specimen by cutting the specimen in half (longitudinally in a sagittal plane) using a slicing blade. Thin sections (5 *μ*m) are cut by a Rotary Microtome (HM 355S, Thermo Fisher Scientific, Inc., Germany) along the long axis of each femur in sagittal plane. HE staining assays was performed according to standard protocols after deparaffinization.

### Bone histomorphometric analysis

Animals were subjected to a scheme of double labeling with intravenous injections of 10mg/Kg Calcein and 0.09mg/g Xylenol at 4-week intervals. After sacrificed, the distracted tibiae were dehydrated in graded concentrations of ethanol and embedded into methyl methacrylate (MMA) with our previously established protocol after microCT analysis. Sagittal sections of distracted tibia in 5 μm was performed with a Leica SM2500E microtome (Leica Microsystems, Germany). The sections were then subjected to Goldner's trichrome staining to analyze bone dynamic histomorphometric parameters, including mineral apposition rate (MAR) and mineral surface/ bone surface (MS/BS) with fluorescence microscopy (Leica image analysis system, Q500MC) and OsteoMeasure system (OsteoMetrics Inc., Decatur, GA, USA). MAR (μm/day) = distance between labels/interlabel period. The bone histomorphometric parameters were calculated and expressed according to the standardized nomenclature for bone histomorphometry.

### Statistical analysis

All quantitative data were transferred to statistical spreadsheets and analyzed by a commercially available statistical program SPSS version 16.0 (IBM, USA); independent *t*-test was used for comparison of mean values with *P* < 0.05 considered as statistically significant.
